# The small bowel diseases detected by capsule endoscopy in patients with chronic abdominal pain: A retrospective study

**DOI:** 10.1097/MD.0000000000010025

**Published:** 2018-02-23

**Authors:** Libin Huang, Zhiyin Huang, Yang Tai, Pu Wang, Bing Hu, Chengwei Tang

**Affiliations:** Department of Gastroenterology, West China Hospital, Sichuan University, Chengdu, China.

**Keywords:** capsule endoscopy, chronic abdominal pain, small bowel disease

## Abstract

Chronic abdominal pain (CAP) remains a particular challenge because of its complicated causes, especially when the disorders involve the small bowel, where it is quite difficult to intubate the flexible endoscopes. This study was to investigate the small bowel diseases detected by capsule endoscopy (CE) in CAP patients to evaluate the role of CE on CAP, and analyzed the relationship among the clinical characteristics of CAP patients and the positive rates of CE findings to search for the indications of CE for CAP patients.

This retrospective study included 341 patients with CAP defined as recurrent abdominal pain for no <3 months. Each patient underwent CE after a negative diagnostic work-up. All CE images were reviewed by 3 gastroenterologists independently. The positive findings were defined as abnormal findings in the small bowel that might have been the causes of CAP. The final diagnosis was confirmed by CE findings, clinical features, histopathology, and a response to the treatment during the follow-up for at least 3 months after CE.

The overall positive rate of CE findings was 28.15% (96/341). The positive rate in CAP-A (CAP with associated symptoms) group was significantly higher than that in CAP-O (CAP only) group (33.16% vs 21.38%, *P* = .017). Multivariate logistic regression analysis revealed that weight loss (odds ratio [OR] = 2.827, 95% confidence interval (CI) = 1.938–4.926), hypoalbuminemia (OR = 6.142, 95%IC = 4.129–8.274), elevated erythrocyte sedimentation rate (ESR) (OR = 4.025, 95%IC = 3.178–6.892), or increased C-reactive protein (CRP) (OR = 7.539, 95%CI = 5.365–11.723) were significantly associated with high positive rates. On follow-up, final diagnosis was confirmed in 56 of 69 (81.16%) patients with positive CE findings. About half of these patients (46.38%, 32/69) were diagnosed as inflammatory diseases, including Crohn disease (12), tuberculosis (5), NSAID enteropathy (4), etc. Tumors were proved in 21.74% (15/69) patients, including malignant in 7 cases and benign in 8 cases. Parasitosis was found in 9 (13.04%) patients.

This study suggests that CE may be helpful for CAP patients to detect the small bowel diseases, half of which were comprised of inflammatory diseases. Besides, weight loss, hypoalbuminemia, elevated ESR, or increased CRP may be regarded as the indications of CE for CAP patients.

## 1 Introduction

Chronic abdominal pain (CAP) is one of the most common complaints, resulting in a poor quality of life for the patients and a high burden of consumption of medical resources.^[1,2]^ CAP has a wide range of possible causes and a variety of mechanisms involved in. Chronic pain stimuli initiated through chemical or mechanical receptors in the intestine are the most important mechanism for CAP. The chemical receptors primarily within the mucosa and submucosa of intestine are directly activated by a variety of chemical substances including prostaglandins, histamine, leukotrienes, and serotonin. These substances can be released in response to inflammation, tissue ischemia, necrosis, or injury, and the accumulation of these substances may also change the microenvironment and result in a reduction in pain threshold.^[3,4]^ The mechanical receptors in the muscular layers can be stimulated by distension of the intestinal tract secondary to mass lesions or stenosis. Sometimes, both chemical and mechanical receptors are involved in CAP. Therefore, the mechanisms and causes of CAP should be quite complicated. In clinical practice, the routine diagnostic work-up for CAP usually includes clinical history, physical examination, laboratory tests, imaging examinations as well as gastroscopy and colonoscopy with biopsies when necessary. However, it is quite difficult to identify its etiologies.^[5–7]^ CAP remains a hard challenge because of its complicated causes, particularly when the disorder involves the small bowel, where it is quite difficult to intubate the flexible endoscopes.

The small bowel has been thought as a “blind area” for physicians because it was quite difficult to be visualized and assessed for many years. In 2001, capsule endoscopy (CE) was approved for endoscopic evaluation of the small bowel by Food and Drug Administration (FDA). Being a simple, noninvasive, and reliable method, CE has been widely used in clinical practice in these years. Accumulating data have demonstrated that CE currently plays an important role in diagnosing the diseases involved in the small bowel, such as obscure gastrointestinal bleeding, suspected Crohn's disease (CD), tumors, celiac disease, and polyposis syndromes.^[8–11]^ However, the limited studies of the diagnostic value of CE for CAP have yielded inconsistent results with a great range of diagnostic yield (9.0–44.4%)^[12–20]^; thus, the value of CE for diagnosing CAP is still unclear. In this study, we retrospectively investigated the findings of CE in CAP patients with a large sample (n = 341) and the final diagnosis of these patients. We aimed to determine the positive rate of CE findings to evaluate the role of CE on CAP. In addition, we analyzed the relationship among the clinical characteristics of CAP patients and the positive rates of CE findings in order to search for the indications of CE for CAP patients.

## 2 Materials and methods

### 2.1 Study design:

This study was approved by the China Ethics Committee of Registering Clinical Trials and registered as ChiCTR-DRD-15006953 in the Chinese Clinical Trial Registry. As a retrospective study, all data were collected from medical records. During this study, there was no trial treatment and no harm to the patients, and patient-identifying information was not part of final analysis, so no consent was required. All data of the patients were reviewed by 3 gastroenterologists who were highly experienced in CE.

### 2.2 Participants

Patients with CAP in this study were from the Department of Gastroenterology, West China Hospital, Sichuan University in the People's Republic of China between January 2007 and November 2015. All data were taken retrospectively from the medical records, including: age, sex, medical history, symptoms, current medications, laboratory tests, imaging studies, as well as the outcomes after CE study.

CAP was defined as recurrent abdominal pain lasting no <3 months from onset. All patients with CAP had undergone a routine diagnostic work-up prior to CE studies, including careful history-taking, physical examination, and laboratory tests which included hemoglobin, fecal occult blood, erythrocyte sedimentation rate (ESR), albumin, and C-reactive protein (CRP). Furthermore esophagogastroduodenoscopy, colonoscopy, and abdominal imaging studies (ultrasonography, barium studies of small bowel, computed tomography, or magnetic resonance imaging) had also been performed for all patients. Unfortunately, there was no evidence to explain the abdominal pain.

Associated symptoms were also recorded, including: chronic diarrhea characterized by recurrent passage of loose/watery stool and/or bowel movement >3 times/d for >6 weeks; weight loss defined as over 10% decrease in body weight within 3 months; distention; nausea; vomiting; constipation; etc.

Patients with gastrointestinal bleeding or positive fecal occult blood testing, diabetes mellitus, organic gastrointestinal diseases, a malignant tumor, or a history of major abdominal surgery were not included. Patients were excluded if the indication for CE examination was abnormal imaging results (i.e., thickening of the small bowel or ulcerations of the small bowel), familial polyposis syndrome, or follow-up of Crohn's disease. Additionally, patients with incomplete data were also excluded.

### 2.3 Capsule endoscopy procedures and findings

The OMOM CE system (Jinshan Science and Technology Company, Chongqing, China) was used for each patient after signing informed consent for CE examination. Patients swallowed the capsule after an overnight fast and bowel preparation with 1600 mL sodium phosphate oral solution in the morning. About 2 to 3 hours later, they were allowed to drink clear fluids and continue their routine daily activities. The patients returned the recording system 8 to 10 hours after the beginning of the examination, and the transmitted video images were downloaded into a computerized system.

All images were reviewed independently by 3 gastroenterologists who were highly experienced in CE and the small bowel diseases. They read all images carefully and made the diagnosis by themselves not blinded to the reason for CE study. In case of discrepancy, the diagnosis was reached on their discussion. The quality of intestinal preparation was determined as excellent (no debris, complete visualization of the mucosa), good (some debris), fair (several areas with incomplete visualization), or poor (large amount of debris that might shade the mucosa). Excellent and good were determined as high quality.

The positive findings in the small bowel were defined as abnormal findings that might have been the cause of abdominal pain, while some findings that might not have contributed to the symptom were excluded, such as red spots and angiodysplasia. The positive rate of CE findings was calculated with the number of patients with positive findings divided by the total number of CAP patients.

### 2.4 The follow-up and final diagnosis

On the basis of the positive CE findings, some interventions were used to identify or treat the patients, including balloon-assisted endoscopic biopsy/resection, surgical resection, and medication. Then the histopathological outcomes for some patients were obtained. Positive response to the treatment was defined as improvement of abdominal pain and/or endoscopic findings on a second CE examination after treatment. The final diagnosis was confirmed by CE findings, clinical features, histopathology, and a response to the treatment during the follow-up for at least 3 months after CE examination.

### 2.5 Statistical analysis

Statistical analysis was performed using the Statistical Package for Social Science (SPSS version 18.0 for Windows, SPSS Inc., Chicago, IL). Descriptive statistics were used to report patients’ demographic and clinical characteristics. Continuous variables were compared using the independent sample *t* test. Categorical variables were compared with the chi-square test. A univariate/multivariate logistic regression analysis was used to analyze the relationships among the clinical characteristics and the positive rate of CE findings. A *P* value <.05 was considered statistically significant.

## 3 Results

### 3.1 Patients with CAP

**Figure 1:**
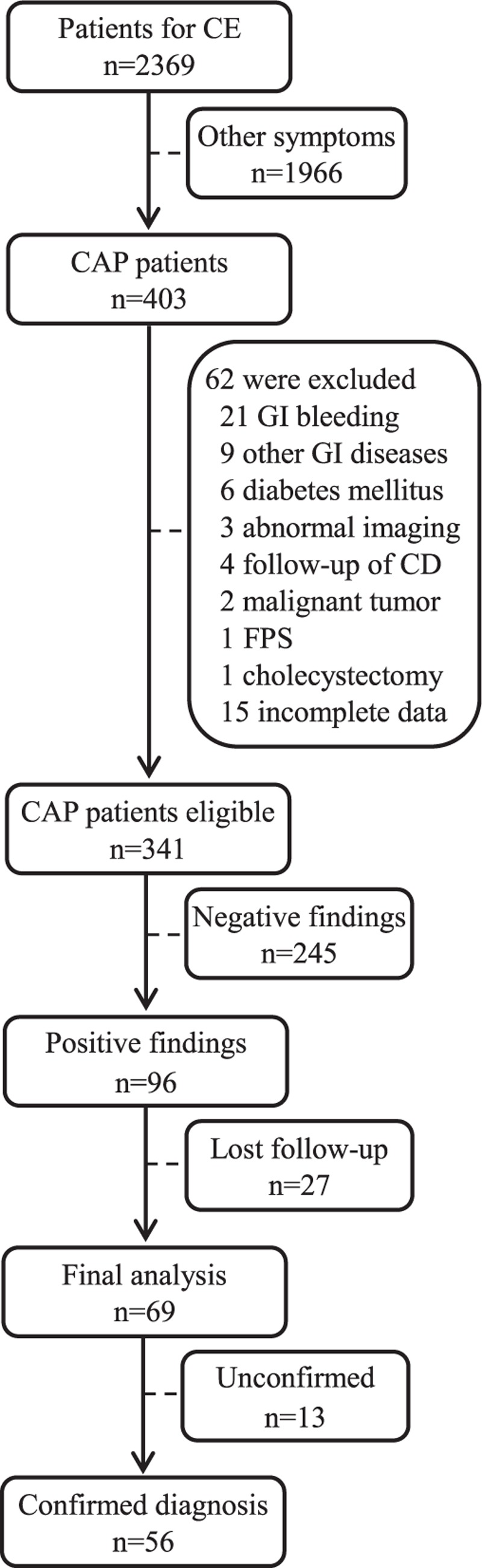
Flow diagram of the patients enrolled in this study. CAP = chronic abdominal pain, CD = Crohn's disease, CE = capsule endoscopy, FPS = familial polyposis syndrome, GI = gastrointestinal.

During the 9-year period, a total of 2369 patients underwent CE examinations. Of these, 403 patients complained of CAP, and 341 cases were included in this retrospective study, the flow diagram was shown in Fig. 1. Of the 341 patients, 163 cases were men and 178 cases were women. The mean age was 44.54 ± 11.61 years (range, 18–75 years). The mean duration of abdominal pain was 5.63 ± 8.52 months (range, 3–240 months). Of the 341 patients, 59 patients complained of mid-epigastric pain, 61 of lower abdominal pain, 136 of periumbilical pain, and 85 of diffuse abdominal pain.

### 3.2 Capsule endoscopy examination

In total, 341 CAP patients underwent 352 CE examinations in this study because a second CE examination was performed in 11 patients during follow-up after the first CE examination. No patient experienced problems in swallowing the capsule. The capsules remained in the stomach over 2 hours in 25 patients and were pushed into the duodenum with the assistance of a snare through gastroscopy. The small bowel transit time was 257.38 ± 88.29 minutes (range, 36–478 minutes). The capsules failed to pass through the ileocecal valves during the video time in 48 patients. The visualization of the whole small intestine identified by passage of ileocecal (IC) valve was achieved in 293 (85.92%) patients. Visualization of the mucosa was excellent in 194 (56.89%), good in 82 (24.05%), fair in 51 (14.96%), and poor in 14 (4.10%). No capsule was found to be retained in patients over 2 weeks, and no other complications were observed.

### 3.3 CE findings and positive rate of CE finding in CAP patients

Positive findings were found in 96 of 341 patients (Table 1), yielding a positive rate of 28.15%. The majority of patients (58/96, 60.42%) presented the inflammatory lesions, including mucosal erosion, ulcers, diverticulitis, and inflammatory stenosis. Parasitosis was found in 9 patients. Mass lesions, such as polyps and tumors, were detected in 29 patients. About half of the lesions (45.83%) occurred in ileum, and one-third presented in both ileum and jejunum. In addition, no positive finding was found in 245 (71.85%) patients.

**Table 1:**
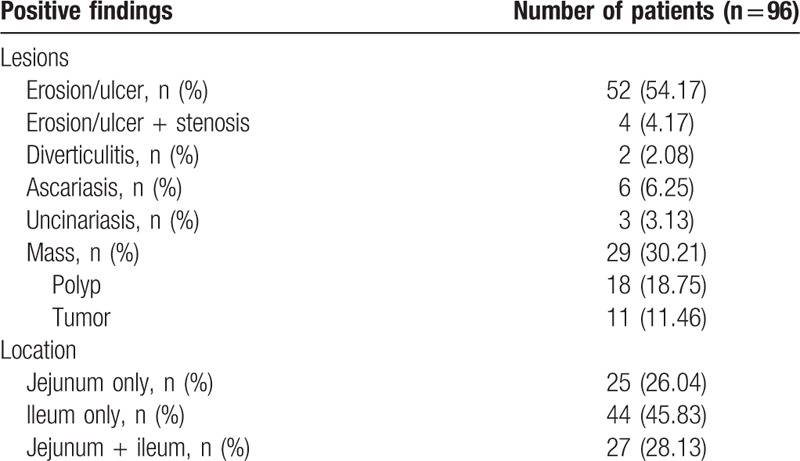
Positive findings of capsule endoscopy in 96 patients with chronic abdominal pain.

### 3.4 The comparison of clinical characteristics and positive rates of CE findings between CAP-A group and CAP-O group

Out of 341 included patients, 196 patients had CAP with associated symptoms (CAP-A), including chronic diarrhea (103), weight loss (65), distension (19), nausea (9), vomiting (7), and constipation (8); while 145 patients had CAP only without associated symptoms (CAP-O). There were no differences on age, sex, duration, and location of abdominal pain between CAP-O and CAP-A, *P* > .05. No differences were shown in the visualization of the whole small intestine and high quality of intestinal preparation between 2 groups, *P* > .05. However, the positive rate of CE findings in CAP-A group (65/196 = 33.16%) was significantly higher than that in CAP-O group (31/145 = 21.38%), *P* = .017 (Table 2).

**Table 2:**
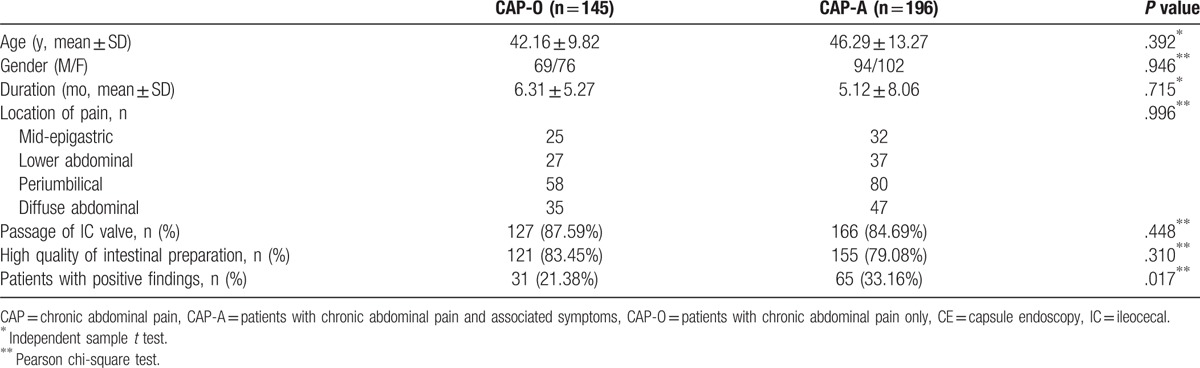
Comparison of characteristics and the positive rates of CE findings between patients with CAP only and patients with CAP and associated symptoms.

### 3.5 The relationships among the clinical characteristics and the positive rates of CE findings in CAP patients

The positive rates of CE findings were significantly higher in CAP patients associated with diarrhea, weight loss, elevated ESR, hypoalbuminemia, or increased C-reactive protein (CRP), *P* < .05 (Table 3). Univariate regression analysis showed that these factors were significantly associated with the positive rates. No relationship was indicated among age, sex, duration, hemoglobin, complete visualization of the whole small intestine and the positive rates. Then multivariate logistic regression analysis revealed that weight loss (odds ratio [OR] = 2.827, 95% confidence interval (CI) = 1.938–4.926), hypoalbuminemia (OR = 6.142, 95%IC = 4.129–8.274), elevated ESR (OR = 4.025, 95%IC = 3.178–6.892), and increased CRP (OR = 7.539, 95%CI = 5.365–11.723) were significantly associated with high positive rates. No significant relationship was found between diarrhea and the positive rate (Table 4).

**Table 3:**
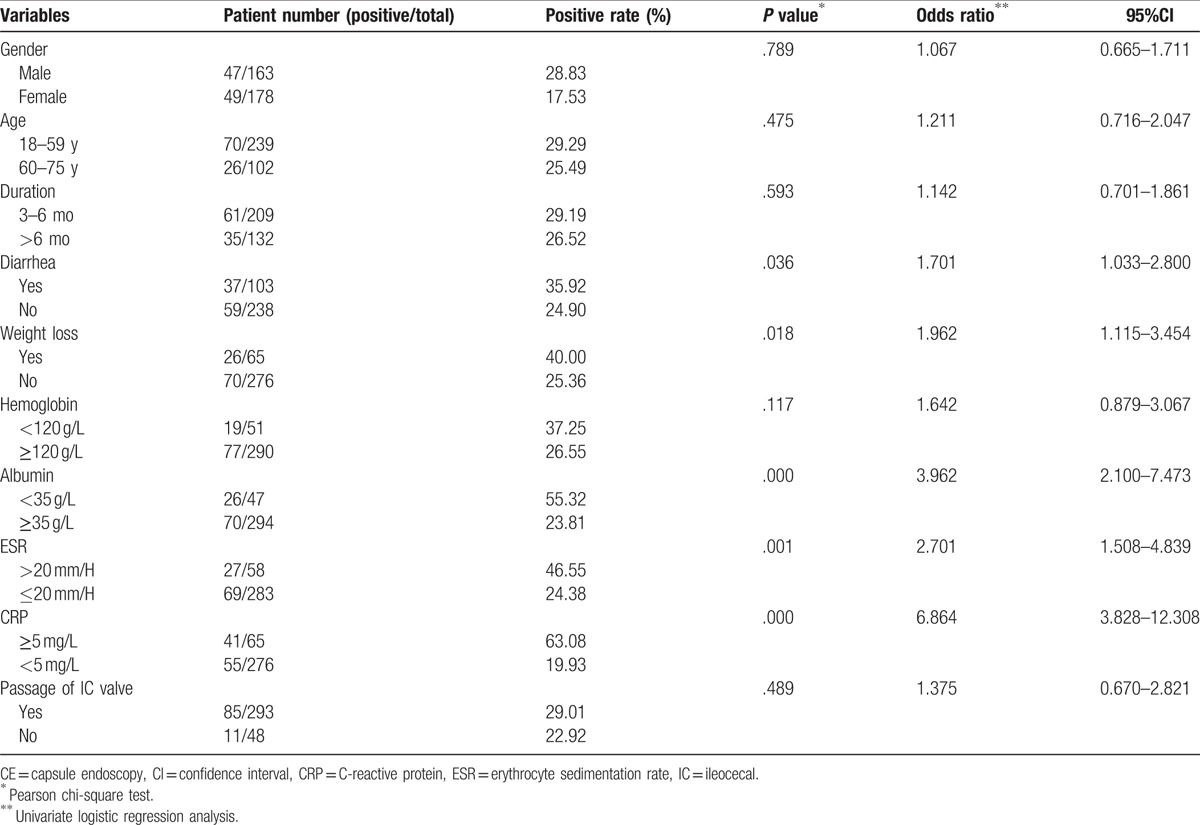
The positive rates of CE findings in subgroups and the relationships among the characteristics and the positive rates by univariate logistic regression analysis.

**Table 4:**
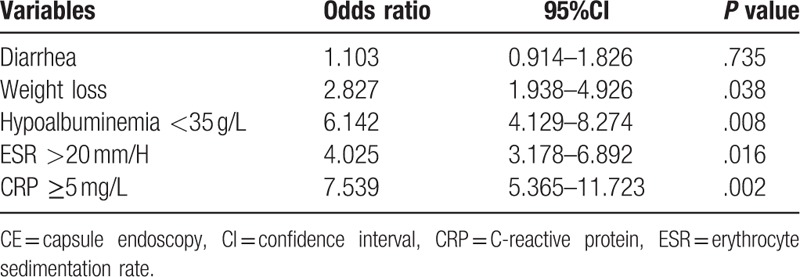
The relationships among the clinical characteristics and the positive rates of CE findings by multivariate logistic regression analysis.

### 3.6 The final diagnosis and outcomes of CAP patients on follow-up

Among 96 CAP patients with positive CE findings, 69 (71.88%) patients had data of follow-up for at least 3 months, other 27 patients were lost. Final diagnosis was confirmed in 56 of 69 patients on the basis of CE findings, clinical features, histopathology, and response to the treatment (Table 5), the diagnostic accuracy rate of CE reached 81.16% (56/69). Then, during 3-month follow-up after CE examination, the majority of patients with confirmed diagnosis (83.93%, 47/56) had improvement of the symptoms.

**Table 5:**
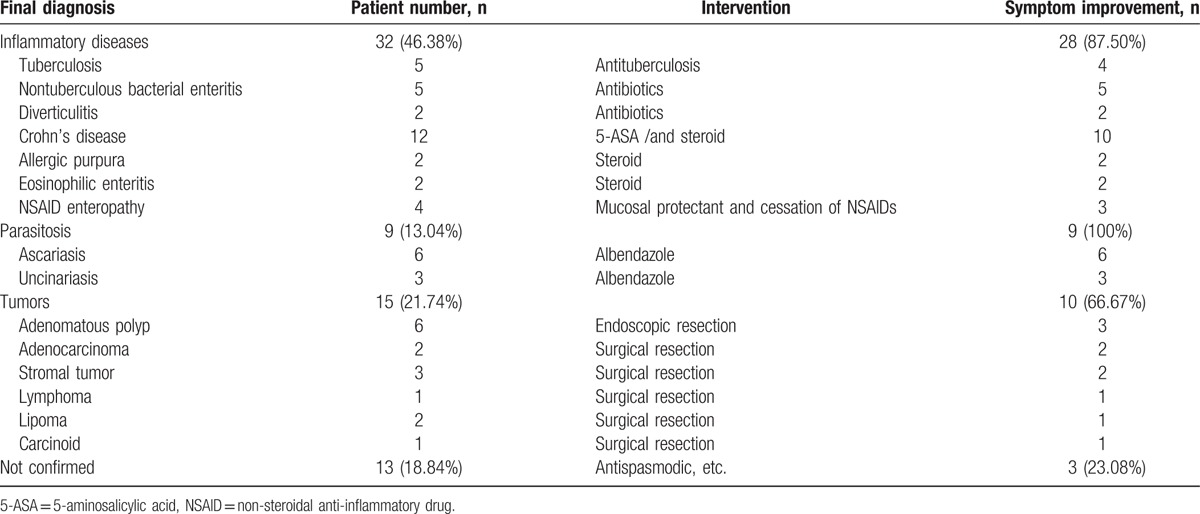
The final diagnosis and outcomes of 69 CAP patients with positive CE findings after 3-month follow-up.

About half of the patients (32/69 = 46.38%) were diagnosed as inflammatory diseases, including CD (12), tuberculosis (5), nontuberculous bacterial enteritis (5), NSAID enteropathy (4), eosinophilic enteritis (2), etc. CD was the most common disease, 10 of 12 CD patients had improvement after treatment with 5-aminosalicylic acid (5-ASA) and/or steroids. Tuberculosis was diagnosed in 5 patients, 4 of whom were judged by histopathology and 1 patient showed a positive response to anti-tuberculosis treatment without histopathological data.

Tumors were proved in 21.74% (15/69) patients by histopathological evidence through endoscopic biopsy/resection or surgery resection, including adenomatous polyp (6), adenocarcinoma (2), stromal tumor (3), etc. The mean age of patients with tumors (n = 15) was 47.23 ± 9.28 years, while the mean age of patients with inflammatory diseases and/or other diseases (n = 41) was 44.83 ± 6.51 years, there was no significant difference between the 2 groups, *P* = .257.

## 4 Discussion

Our results showed an overall quite high positive rate (28.15%) of CE findings in small bowel in CAP patients. In literatures, there were limited studies on the diagnostic value of CE for CAP showing a wide range of yield from 9.0% to 44.4%. Most of the studies had a small sample size (<100 patients) which might have concealed the value of CE.^[12–18]^ Only 2 studies included >100 patients, and their diagnostic yield were 17.3% (19/110) and 23.0% (56/243), respectively.^[19,20]^ Recently, a systemic review reported that the pooled diagnostic yield of CE in CAP was 20.9%.^[21]^ In our study, 341 CAP patients were included and the positive rate (28.15%) was quite higher than the previous studies which had >100 participants.^[19,20]^ The difference might be relevant to our large sample size and different proportion of CAP-O and CAP-A patients. More patients presented positive findings of CE in CAP-A group than CAP-O group in our study, similar results were reported by Egnatios et al.^[17]^

However, the positive rates of CE findings from our study and other previous studies were not as high as those in patients with obscure gastrointestinal bleeding (OGIB) reported recently (49.1–66.9%).^[22–24]^ Actually, it has been well accepted that esophagogastroduodenoscopy and colonoscopy have important role in diagnosing gastrointestinal diseases in spite of their diagnostic yields reported as 23–46% and 24%, respectively.^[25,26]^ On the other hand, patients with CAP lasting >6 months are subjected to be judged as functional gastrointestinal diseases (FGIDs) when conventional diagnostic work-up shows an absence of organic disorders.^[27,28]^ In this study, 132 patients suffered from CAP over 6 months, 26.52% (35/132) of them presented positive findings of small bowel on CE images. It was indicated that FGIDs should be diagnosed cautiously without CE, and the value of CE for CAP might not be denied so as to reduce the misdiagnosis.

In our study, the positive rate of CE findings in CAP-A group was significantly higher than that in CAP-O group (33.16% vs 21.38%, *P* = .017). It seemed that associated symptoms might increase the positive rate of CE findings. Then the relationship among the clinical characteristics and the positive rates were analyzed by univariate and multivariate logistic regression analysis. The results showed that elevated ESR and increased CRP were significantly associated with high positive rates. This result was similar to that from a Greek multicenter study reported by Katsinelos et al.^[16]^ Being inflammatory markers, ESR and CRP play important role in diagnosis and treatment of inflammatory diseases, such as inflammatory bowel disease (IBD), tuberculosis, etc.^[29,30]^ Studies have demonstrated that CRP might be useful for evaluating activity of CD.^[31–33]^ About half of CAP patients (32/69 = 46.38%) in this study were diagnosed as the inflammatory diseases. Among these diseases, CD was the most common one. It was reported that approximately 30% of early CD only involved the small bowel.^[34,35]^ Without CE evaluation, these lesions might be overlooked leading to a delay in diagnosis. CE might play an important role in diagnosing early CD only involved the small bowel. In addition, relationship was shown between hypoalbuminemia and the positive rate in our results. As we know, the mucosal lesions in the intestine might cause protein-losing continuously leading to the complication of hypoalbuminemia. Our results suggested that hypoalbuminemia, elevated ESR, or increased CRP might be the indications of CE for CAP patients suffered from CD.

As we know, weight loss is a nonspecific symptom which may occur in a variety of diseases as a consequence of chronic organic disorders or cachexia. In our study, both univariate and multivariate regression analysis showed weight loss was significantly associated with the positive rate. Similarly, the result that weight loss increased the diagnostic yield of CE was reported in a Korean multicenter study.^[19]^ While in a prospective multicenter trial the result showed that there was no relationship between weight loss and the positive CE findings.^[15]^ The controversial results might be related to the small sample size in both previous studies (n = 110, n = 50, respectively) and fewer patients suffering from weight loss (n = 6, n = 38, respectively). Additionally, there was no definition of weight loss in the first study. So studies with high quality are needed to verify the relationship between weight loss and the positive rate of CE in future. In clinical practice, weight loss is usually regarded as a warning signal which suggests further examinations to search for its causes and intensive care. Our results suggested that weight loss might be regarded as an indication of CE for CAP patients.

The tumors in the small bowel are difficult to detect because of the limitations of intestinal investigation. Patients with tumors in the small bowel typically maintain asymptomatic for years or present only nonspecific symptoms such as abdominal pain.^[36]^ In our study, 15 out of 69 (21.74%) CAP patients were diagnosed as tumors, including malignant in 7 cases and benign in 8 cases. In a systematic review included 290 patients with unexplained abdominal pain from 15 papers, tumors in the small bowel were found in 9.0% (26/290) patients.^[21]^ The difference of the constituent ratio of tumors might be associated with relatively high heterogeneity between 2 studies, such as the definition of chronic abdominal pain and unexplained abdominal pain, the clinical characteristics of the included patients, the physicians who read the images of CE and made the diagnosis. Our results suggested that the tumors in the small bowel should not be ignored in CAP patients, and CE can play an important role on detecting the lesions. Additionally, our results showed that there was no significant difference on age between the patients with tumors and the patients with inflammatory diseases and/or other diseases. However, the number of patients with tumors in this study was quite fewer (n = 15), so the difference in terms of age should be re-evaluated by enlarged sample size in future studies.

The small bowel is difficult to be thoroughly examined because of its length, location, and tortuosity. Currently, radiographic imaging still plays a pivotal role in the examination of the small bowel.^[37]^ Barium studies have been utilized clinically for many years as a traditional mode of examination despite its low sensitivity. Computed tomography enteroclysis (CTE) and magnetic resonance imaging enterography (MRIE) have been recommended as first-line modalities.^[38]^ Compared with CTE and MRIE, CE might have high sensitivity to the mucosal lesions, such as erosions and superficial ulcers which are difficult to be shown on radiographic imaging. On the other hand, push enteroscopy and balloon-assisted endoscopy have been used for examining the small bowel in the present decade.^[39]^ Actually they are restricted in clinical practice because of their invasiveness, patient intolerance of the procedure, and physicians’ difficulty in operating.^[40]^ Unlike these modalities, CE as a simple and non-invasive method showing direct and clear images, may be more convenient and effective in diagnosing disorders in the small bowel. In this study, although only 28.15% (96/341) CAP patients presented positive findings through CE, 81.16% (56/69) patients with positive CE findings had a confirmed diagnosis on follow-up, and 83.93% of these patients had improvement of abdominal pain after CE examination. The results showed that CE might play a valuable role in diagnosis and improvement of the symptoms for CAP patients.

Although CE shows relatively low specificity and an inability to confirm the nature of detected lesions histologically, the subsequent series of measures such as balloon-assisted endoscopy with biopsy, serological tests, and diagnostic therapy with 5-ASA or antitubercular agents may contribute to establishing a final diagnosis for these patients. On the other hand, CE should be restrictive to use for diagnosing CD because it was reported that CE retention occurred in up to 13.2% of the patients with suspected or confirmed CD.^[41]^ In this study, no capsule retention was observed, even though stenosis was shown on CE images in 4 patients. It indicates that CE would be safe for CAP patients through conventional screening to exclude patients with intestinal stricture.

Of course, the present study does have some limitations on its retrospective design. Although patients with incomplete data were excluded in the process of inclusion, the information of the participants in the study might be imperfect. For example, some patients in CAP-O group might have suffered from some associated symptoms which were not presented in their medical records, and they might be divided into CAP-A group in fact. Additionally, we did not have follow-up data for all of the participants who underwent CE for CAP. Of 96 patients who showed positive CE findings, 27 (28.13%) patients were lost to follow-up and their final diagnosis was unknown. The loss of follow-up might lead to the deficiency of determined diagnosis and outcomes after CE. Therefore, a controlled prospective study is necessary to further evaluate the role of CE on CAP.

In conclusion, this study suggested that CE should be helpful for CAP patients to detect the small bowel diseases, half of which were comprised of inflammatory diseases. Besides, weight loss, hypoalbuminemia, elevated ESR or increased CRP may be regarded as the indications of CE for CAP patients.

## Acknowledgments

The authors would like to thank Dr. Jiantong Shen (from The Chinese Cochrane Center) who helped to design the methods of statistics analysis and review the manuscript.
